# Amino Acid Substitutions at P1 Position Change the Inhibitory Activity and Specificity of Protease Inhibitors BmSPI38 and BmSPI39 from *Bombyx mori*

**DOI:** 10.3390/molecules28052073

**Published:** 2023-02-22

**Authors:** Youshan Li, Meng Wei, Jie Zhang, Rui Zhu, Yuan Wang, Zhaofeng Zhang, Changqing Chen, Ping Zhao

**Affiliations:** 1 College of Biological Science and Engineering, Shaanxi University of Technology, Hanzhong 723001, China; 2 Qinba Mountain Area Collaborative Innovation Center of Bioresources Comprehensive Development, Hanzhong 723001, China; 3 Qinba State Key Laboratory of Biological Resources and Ecological Environment (Incubation), Shaanxi University of Technology, Hanzhong 723001, China; 4 Shaanxi Province Key Laboratory of Bio-Resources, Hanzhong 723001, China; 5 State Key Laboratory of Silkworm Genome Biology, Southwest University, Chongqing 400715, China

**Keywords:** protease inhibitor, elastase inhibitor, amino acid substitutions, inhibitory activity, inhibitory specificity, *Bombyx mori*

## Abstract

It was found that silkworm serine protease inhibitors BmSPI38 and BmSPI39 were very different from typical TIL-type protease inhibitors in sequence, structure, and activity. BmSPI38 and BmSPI39 with unique structure and activity may be good models for studying the relationship between the structure and function of small-molecule TIL-type protease inhibitors. In this study, site-directed saturation mutagenesis at the P1 position was conducted to investigate the effect of P1 sites on the inhibitory activity and specificity of BmSPI38 and BmSPI39. In-gel activity staining and protease inhibition experiments confirmed that BmSPI38 and BmSPI39 could strongly inhibit elastase activity. Almost all mutant proteins of BmSPI38 and BmSPI39 retained the inhibitory activities against subtilisin and elastase, but the replacement of P1 residues greatly affected their intrinsic inhibitory activities. Overall, the substitution of Gly54 in BmSPI38 and Ala56 in BmSPI39 with Gln, Ser, or Thr was able to significantly enhance their inhibitory activities against subtilisin and elastase. However, replacing P1 residues in BmSPI38 and BmSPI39 with Ile, Trp, Pro, or Val could seriously weaken their inhibitory activity against subtilisin and elastase. The replacement of P1 residues with Arg or Lys not only reduced the intrinsic activities of BmSPI38 and BmSPI39, but also resulted in the acquisition of stronger trypsin inhibitory activities and weaker chymotrypsin inhibitory activities. The activity staining results showed that BmSPI38(G54K), BmSPI39(A56R), and BmSPI39(A56K) had extremely high acid–base and thermal stability. In conclusion, this study not only confirmed that BmSPI38 and BmSPI39 had strong elastase inhibitory activity, but also confirmed that P1 residue replacement could change their activity and inhibitory specificity. This not only provides a new perspective and idea for the exploitation and utilization of BmSPI38 and BmSPI39 in biomedicine and pest control, but also provides a basis or reference for the activity and specificity modification of TIL-type protease inhibitors.

## 1. Introduction

Proteases exist widely in organisms, and participate in the decomposition of proteins to maintain the normal life activities of organisms. Protease inhibitors are the main regulators of protease catalytic activity in vivo, which can bind protease molecules and inhibit their physiological activity, thereby terminating the unwanted proteolytic process [1]. The interaction between protease and inhibitor is highly specific, that is, each protease has its specific inhibitor as its regulatory factor. The location and activity of these protease inhibitors greatly affect the function of the corresponding proteases, and thus determine the fate of various tissues and cells at specific stages of growth and development. The mutation of protease inhibitors will affect their biological effects. The analysis of the key active sites of protease inhibitors will not only deepen people’s understanding of their active mechanism of action, but is also conducive to their bioengineering transformation and application [2].

The silkworm, *Bombyx mori*, is a silk-spinning insect with important economic value, which has accumulated a large amount of basic research, and has become one of the ideal models of Lepidoptera in the fields of the biochemistry, genetics, and genomics [3,4,5,6]. Unlike mammals, insects lack lymphocytes or immunoglobulins, and serine protease inhibitors are thought to play important roles in insect immunity [7,8,9]. In our previous study, 80 serine protease inhibitor (SPI) genes were identified from the silkworm genome sequence, named BmSPI1 to BmSPI80, respectively. Ten SPI domains were identified from these serine protease inhibitors, including Serpin, Kunitz, Kazal, Trypsin inhibitor-like cysteine-rich (TIL), Amfpi, Bowman-Birk, Antistasin, WAP, Pacifast, and Alpha-macroglobulin [10]. Further studies revealed that serine protease inhibitors BmSPI38 and BmSPI39 have a unique TIL domain, which can inhibit the activity of the virulence protease CDEP-1 from *Beauveria bassiana*, inhibit the germination of *B. bassiana* conidia, and thus enhance the antifungal capacity of silkworm [11,12]. Protease inhibitors are abundant in silkworm silk. TIL family protease inhibitors, represented by BmSPI39, can enter the cocoon layer during the process of silk secretion, endow the cocoon with strong anti-microbial protease hydrolysis properties, and provide long-term protection for the development of pupa in the cocoon [13,14].

The TIL domain of most TIL family protease inhibitors in nature usually contains 10 conserved cysteine residues, forming 5 intramolecular disulfide bonds. The disulfide bridge positions are 1–7, 2–6, 3–5, 4–10, and 8–9 [15,16,17]. Such typical TIL-type protease inhibitors mainly inhibit cathepsin, trypsin, chymotrypsin, and other mammalian proteases [18,19,20]. However, our previous studies found that the TIL domain of BmSPI38 and BmSPI39 lack the second and sixth cysteine residues, which can strongly inhibit the activities of microbial proteases such as subtilisin, proteinase K, and *Aspergillus melleus* protease, but have no inhibitory effect on trypsin and chymotrypsin [11,12]. It was suggested that the absence of Cys^2nd^ and Cys^6th^ may be the important reason for them to obtain inhibitory activity against microbial proteases. Cys^2nd^ and Cys^6th^ were introduced into the corresponding sites of BmSPI38 and BmSPI39 by site-directed mutagenesis. The results showed that the introduction of cysteines not only affected the polymerization state of BmSPI38 and BmSPI39, but also significantly reduced their inhibitory activity against microbial proteases [21]. Unfortunately, the introduction of Cys^2nd^ and Cys^6th^ did not change the inhibitory specificity of BmSPI38 and BmSPI39. By systematically summarizing the sequence characteristics and activities of reported TIL family protease inhibitors, it is found that the inhibitory specificity of these inhibitors may have a certain rule. The inhibitory activity and specificity of TIL family protease inhibitors may be determined jointly by the two missing cysteines (Cys^2nd^ and Cys^6th^), and the physicochemical properties of P1 and P1′ residues in the reactive center [21]. However, the above speculation still needs more experimental evidence to support it.

It is well known that small-molecule serine protease inhibitors bind to proteases as substrate analogues, and at the same time the peptide bond in their reactive center is cleaved. Previous studies have shown that P1 residues in the reactive center of serine protease inhibitors largely determine their inhibitory specificity, and any change in P1 residues may affect the specificity and inhibitory ability of the inhibitor [22,23,24,25,26]. So far, the activity and function of BmSPI38 and BmSPI39 have been relatively clear, but the mechanism of action is not fully understood, and the key sites affecting their inhibitory activity and specificity need to be further explored.

In this study, mutant proteins were obtained by site-directed mutagenesis and prokaryotic expression techniques, and the effects of amino acid substitutions at the P1 position on the inhibitory activity and specificity of protease inhibitors BmSPI38 and BmSPI39 were investigated in vitro. This was carried out in order to provide a basis or reference for the inhibitory activity and specificity modification of TIL family protease inhibitors, and to promote the production and application of such inhibitors.

## 2. Results

### 2.1. BmSPI38 and BmSPI39 Have Inhibitory Activity against Elastase

**Figure 1:**
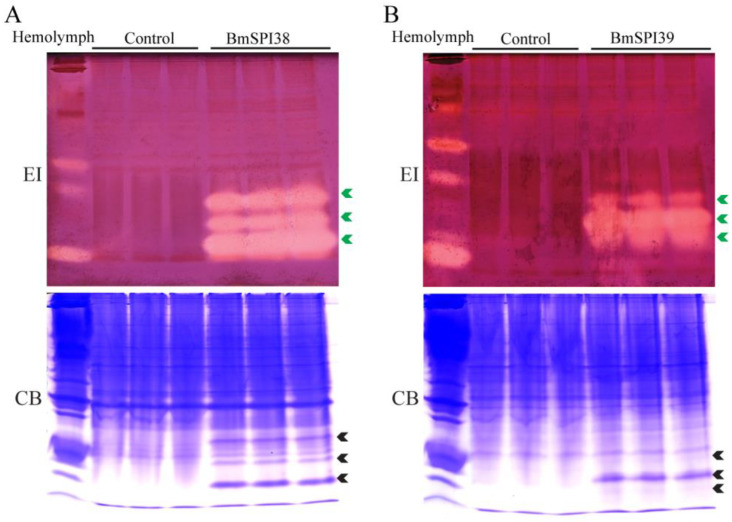
BmSPI38 and BmSPI39 have elastase inhibitory activity. Activity staining of recombinantly expressed BmSPI38 (**A**) and BmSPI39 (**B**) against elastase. The soluble *E. coli* extracts containing the recombinant BmSPI38 or BmSPI39 were separated with 10% Native PAGE and visualized by in-gel activity staining against elastase. *B. mori* hemolymph from day 5 fifth-instar larvae was used as positive control. “Control” represents the lysate supernatant of Origami 2(DE3) or BL21(DE3) cells transformed with the p28 plasmid. “EI” indicates inhibitory activity against elastase, and “CB” indicates protein staining with Coomassie Brilliant Blue R250. Green arrows indicate the active bands of the protease inhibitors, and black arrows indicate the Coomassie Brilliant Blue-stained protein bands at the position corresponding to the inhibitory-active bands.

It was found that elastase has a strong hydrolysis ability for silk fibroin protein, and its hydrolysis ability was significantly stronger than that of trypsin, chymotrypsin, papain, collagenase, and alkaline protease, which may be due to the large number of hydrolysis sites of elastase in silk fibroin protein [27,28,29,30]. To explore whether BmSPI38 and BmSPI39, which are highly expressed in the silk glands of *B. mori*, have elastase inhibitory activity, an in-gel activity staining technique was used to examine the inhibitory activity of recombinant BmSPI38 and BmSPI39 proteins towards porcine pancreatic elastase. Three strong inhibitory-active bands were detected for both recombinant BmSPI38 and BmSPI39, indicating that they could strongly inhibit elastase activity (Figure 1A,B). This is the first time that the inhibitory activity of silkworm protease inhibitors against elastase has been defined.

### 2.2. Design, Expression Vector Construction, and Prokaryotic Expression of the P1 Mutants of BmSPI38 and BmSPI39

**Figure 2:**
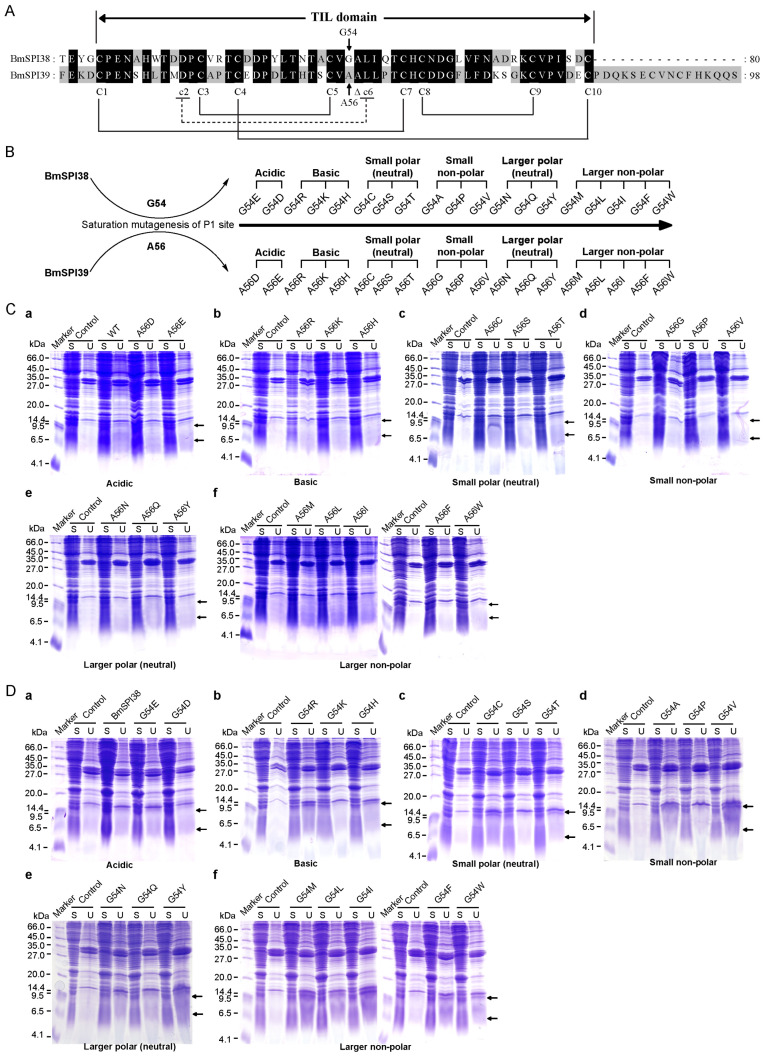
Design, expression vector construction and prokaryotic expression of the P1 mutants of BmSPI38 and BmSPI39. (**A**) The P1 residues and the conserved cysteines in the TIL domain of BmSPI38 and BmSPI39. The eight conserved cysteines are shown with capital C. “c2” and “c6” indicate the second and sixth conserved cysteines missing from the TIL domain of BmSPI38 and BmSPI39, respectively. The four intramolecular disulfide bonds formed by conserved cysteines are shown as black solid lines. The missing disulfide bond between Cys^2nd^ and Cys^6th^ is shown as a dashed line. The P1 and P1′ residues in the reactive centers are indicated by arrows and empty triangles, respectively. Gly54 and Ala56 are putative P1 residues of BmSPI38 and BmSPI39, respectively. (**B**) Schematic representation of saturation mutagenesis at P1 position of BmSPI38 and BmSPI39. (**C**) Prokaryotic expression of the P1 mutants of BmSPI39. SDS-PAGE analysis of BmSPI39 mutants with acidic (**a**), basic (**b**), small polar (neutral) (**c**), small non-polar (**d**), larger polar (neutral) (**e**), and larger non-polar (**f**) amino acids at P1 position. The theoretical molecular weights of the monomer and dimer of the BmSPI39 mutant proteins are about 9.3 kDa and 18.6 kDa, respectively. The arrows show the recombinant BmSPI39 mutant proteins. The upper arrow indicates the band corresponding to the dimer of the BmSPI39 mutant protein and the lower arrow indicates the band corresponding to its monomer. (**D**) Prokaryotic expression of the P1 mutants of BmSPI38. SDS-PAGE analysis of BmSPI38 mutants with acidic (**a**), basic (**b**), small polar (neutral) (**c**), small non-polar (**d**), larger polar (neutral) (**e**), and larger non-polar (**f**) amino acids at P1 position. The theoretical molecular weights of the monomer and dimer of the BmSPI38 mutant proteins are about 7.5 kDa and 15 kDa, respectively. The arrows show the recombinant BmSPI38 mutant proteins. The upper arrow indicates the band corre-sponding to the dimer of the BmSPI38 mutant protein and the lower arrow indicates the band corresponding to its monomer.

As shown in Figure 2A, the TIL domains of BmSPI38 and BmSPI39 contain eight conserved cysteines that form four intramolecular disulfide bridges. In contrast to typical TIL-type protease inhibitors, BmSPI38 and BmSPI39 lack the second and sixth cysteines. It was noted that Cys^2nd^ and Cys^6th^ formed exactly a disulfide bond bridge in the typical TIL domain [15,16,17]. The putative P1 residues in the reactive centers of BmSPI38 and BmSPI39 are Gly54 and Ala56, respectively. In order to explore the effects of P1 residues on the inhibitory activity and specificity of BmSPI38 and BmSPI39, saturation mutagenesis was performed on Gly54 in BmSPI38 and Ala56 in BmSPI39 by utilizing site-directed mutagenesis (Figure 2B). Based on amino acid properties, The P1 residues in BmSPI38 and BmSPI39 were replaced by acidic (Glu and Asp), basic (Arg, Lys and His), small polar neutral (Cys, Ser and Thr), small non-polar (Ala/Gly, Pro, and Val), larger polar neutral (Asn, Gln and Tyr), and larger non-polar amino acids (Met, Leu, Ile, Phe, Trp), respectively.

To obtain active recombinant mutant proteins for subsequent studies, P1-mutant expression vectors of BmSPI38 and BmSPI39 were transformed into *E. coli* Origami 2(DE3) and BL21(DE3) cells for induced expression, respectively. The P1 mutant proteins recombinantly expressed in *E. coli* cells were separated and detected by 16.5% reducing SDS-PAGE. The results showed that the P1 mutant proteins of BmSPI39 were mainly expressed in soluble form in BL21(DE3) cells, and obvious protein expression bands were detected at the size of its monomer (about 9.3 kDa) and dimer (about 18.6 kDa) (Figure 2C). All P1 mutant proteins of BmSPI38 were detected in the supernatant of Origami 2(DE3) cell lysate, and their apparent molecular weights were basically consistent with their monomers (about 7.5 kDa) and dimers (about 15 kDa) sizes (Figure 2D). These results indicated that folded or functional P1 mutants of BmSPI38 and BmSPI39 could be obtained by prokaryotic expression technology.

### 2.3. Inhibition Activity of the P1 Mutants of BmSPI38 against the Serine Protease

**Figure 3:**
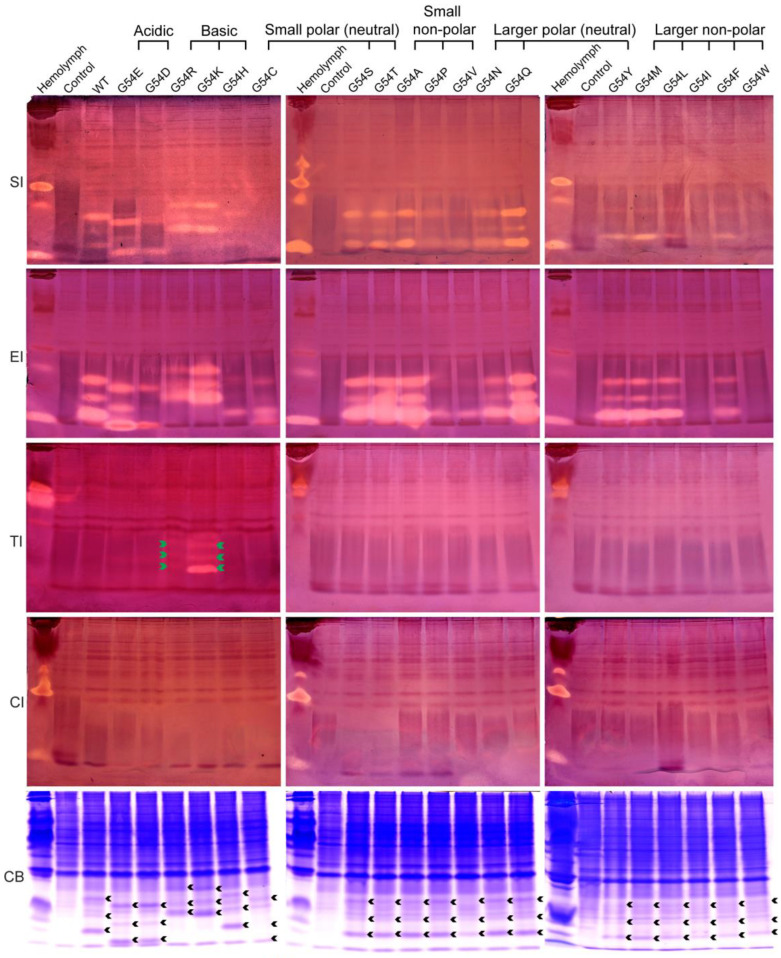
Activity staining of the P1 mutants of BmSPI38 against the serine protease. *B. mori* hemolymph from day 5 fifth-instar larvae was used as positive control. “Control” represents the lysate supernatant of Origami 2(DE3) cells transformed with the p28 plasmid. “WT” represents the wild-type recombinant BmSPI38 protein. “SI”, “EI”, “TI” and “CI” indicate inhibitory activity against subtilisin, elastase, trypsin, and chymotrypsin, respectively. “CB” indicates protein staining with Coomassie Brilliant Blue R250. Green arrows indicate the inhibitory-active bands of the P1 mutants against trypsin, and black arrows indicate the Coomassie Brilliant Blue-stained protein bands at the position corresponding to the inhibitory-active bands.

To investigate the effect of amino acid substitutions at the P1 position on the inhibitory activity and specificity of BmSPI38, we analyzed the inhibitory activity of these mutants against subtilisin, elastase, trypsin, and chymotrypsin using in-gel activity staining of protease inhibitor (Figure 3). Combined with the relative expression levels of the mutant proteins in *E. coli* cells (Figure 3, CB), the inhibitory activities of these mutants against different serine proteases were preliminarily compared according to the strength of the inhibitory-active bands. All mutants, except G54C, G54I, and G54W, showed inhibitory activities against subtilisin. All the mutants exhibited inhibitory activity against elastase, but the inhibitory activities of G54I and G54W were extremely weak. The inhibitory activity of G54C was only detected against elastase, but not against subtilisin. The inhibitory activities of G54Q, G54S, G54T, and G54A against subtilisin and elastase were significantly stronger than those of wild-type BmSPI38. The inhibitory activities against subtilisin were roughly G54C, G54I, G54W << G54H, G54P, G54V < wild type, while the inhibitory activities against elastase were roughly G54I, G54W << G54P, G54V, G54H < G54C, G54D < wild type. When P1 residues were replaced with strong basic amino acids Arg or Lys, G54R and G54K not only retained strong inhibitory activities against subtilisin and proteinase K, but also gained inhibitory activities against trypsin. The inhibitory activity of G54K towards trypsin was stronger than that of G54R. All mutants except G54R and G54K failed to inhibit trypsin activities. None of the mutants showed an inhibitory activity against chymotrypsin. These results indicate that the replacement of P1 residues not only affects the intrinsic activities of BmSPI38 (the inhibitory activities against subtilisin and elastase), but also changes its inhibitory specificity by replacing Gly with Arg or Lys.

### 2.4. Inhibition Activity of the P1 Mutants of BmSPI39 against the Serine Protease

**Figure 4:**
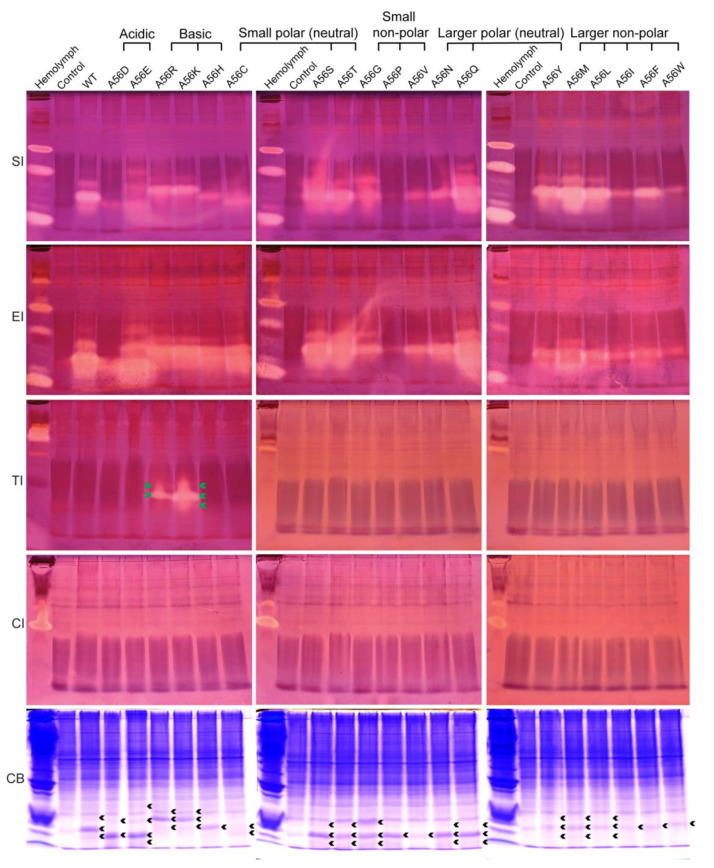
Activity staining of the P1 mutants of BmSPI39 against the serine protease. *B. mori* hemolymph from day 5 fifth-instar larvae was used as positive control. “Control” represents the lysate supernatant of Origami 2(DE3) cells transformed with the p28 plasmid. “WT” represents the wild-type recombinant BmSPI39 protein. “SI”, “EI”, “TI” and “CI” indicate inhibitory activity against subtilisin, elastase, trypsin, and chymotrypsin, respectively. “CB” indicates protein staining with Coomassie Brilliant Blue R250. Green arrows indicate the inhibitory-active bands of the P1 mutants against trypsin, and black arrows indicate the Coomassie Brilliant Blue-stained protein bands at the position corresponding to the inhibitory-active bands.

Similarly, the effects of amino acid replacements at the P1 site on the intrinsic activity and inhibitory specificity of BmSPI39 were explored by using in-gel activity staining technology (Figure 4). The results showed that all mutant proteins could inhibit the activities of subtilisin and elastase, but not chymotrypsin. Overall, the inhibitory activities of A56Q, A56S, and A56T against subtilisin and elastase were significantly stronger than those of wild-type BmSPI39, while the inhibitory activities of A56D, A56P, A56V, A56I, and A56W were significantly weaker than those of wild-type BmSPI39. Although A56Y, A56M, and A56L showed little difference in elastase inhibitory activity compared to wild-type BmSPI39, their subtilisin inhibitory activity was significantly stronger than wild-type BmSPI39. When P1 residues were replaced by Arg or Lys, the mutant proteins not only retained strong inhibitory activities against subtilisin and elastase, but also acquired the trypsin inhibitory activity. Similar to the results of BmSPI38, BmSPI39(A56K) showed stronger inhibitory activity against trypsin than BmSPI39(A56R). In conclusion, the replacements of P1 residues not only greatly affect the intrinsic activity of BmSPI39, but also change the inhibitory specificity of the mutants when P1 residues are replaced with strong basic amino acids.

### 2.5. The Replacement of P1 Residue with Lysine or Arginine Enables BmSPI38 and BmSPI39 to Obtain Trypsin Inhibitory Activity

**Figure 5:**
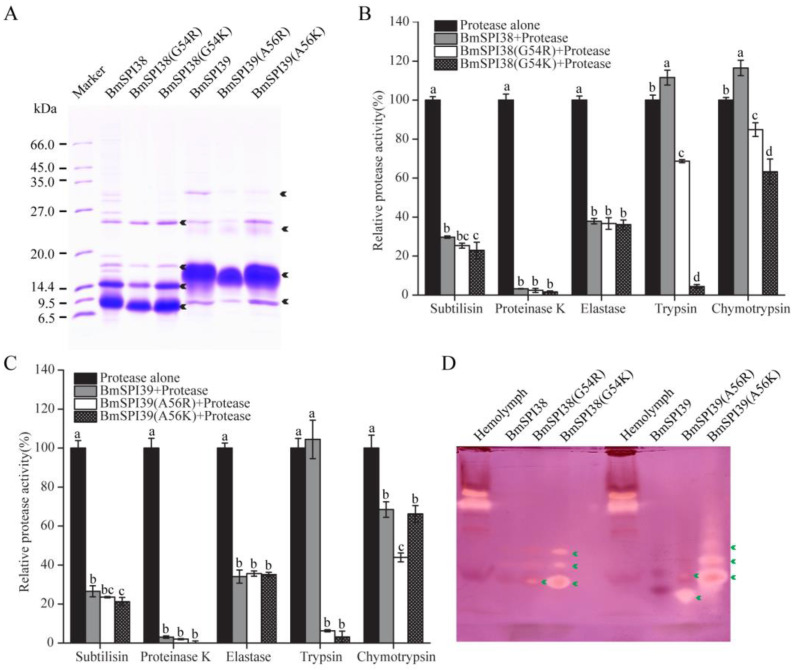
The replacement of P1 residue with lysine or arginine enables BmSPI38 and BmSPI39 to obtain trypsin inhibitory activity. (**A**) Purification of wild-type and mutant protease inhibitors. Arrowheads indicate different multimerized forms of the wild-type and mutant proteins. The inhibitory activities of mutant BmSPI38 (**B**) and BmSPI39 (**C**) proteins towards different serine proteases using protease inhibition assays. Error bars represent the standard error of the mean (*n* = 3). Different letters “a–d” indicate a significant difference between groups (*p* < 0.05), and one identical letter indicates no significant difference between groups (*p* < 0.05). (**D**) Activity staining of purified mutant proteins towards trypsin. *B. mori* hemolymph from day 5 fifth-instar larvae was used as positive control. Green arrows indicate the inhibitory-active bands of the P1 mutants against trypsin.

To further confirm the inhibitory activities of the mutant proteins with a strong basic amino acid at the P1 position against serine proteases represented by trypsin, recombinant BmSPI38, BmSPI38(G54R), BmSPI38(G54K), BmSPI39, BmSPI39(A56R), and BmSPI39(A56K) were purified by immobilized-nickel affinity chromatography. In addition to the monomer forms of wild-type and mutant proteins, the dimers, trimers, and a small amount of higher-order multimeric forms were also detected in SDS-PAGE (Figure 5A). Protease inhibition assays confirmed that BmSPI38(G54R), BmSPI38(G54K), BmSPI39(A56R), and BmSPI39(A56K) not only retained strong inhibitory activities against subtilisin, proteinase K, and elastase, but also obtained strong inhibitory activities against trypsin (Figure 5B,C). In general, the mutants with Lys at P1 have stronger inhibitory activity against trypsin than that of Arg. In-gel activity staining of P1 mutant proteins further confirmed the inhibitory activity of BmSPI38(G54R), BmSPI38(G54K), BmSPI39(A56R), and BmSPI39(A56K) against trypsin (Figure 5D). Notably, BmSPI38(G54R) and BmSPI38(G54K) also achieved a certain degree of chymotrypsin inhibitory activities compared with wild-type BmSPI38 (Figure 5B). Unexpectedly, BmSPI39 also showed weak chymotrypsin inhibitory activity, which was somewhat enhanced by alkaline amino acid substitution (Figure 5C).

### 2.6. Comparison of the Inhibitory Ability of Mutant BmSPI38 Proteins to Different Serine Proteases

**Figure 6:**
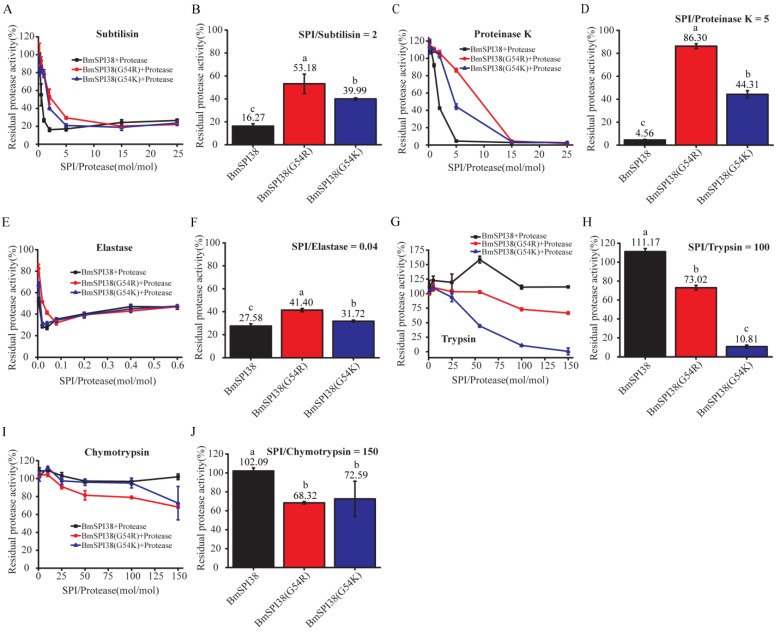
Comparison of the inhibitory ability of mutant BmSPI38 proteins to different serine proteases. Inhibitory effects of increasing concentrations of mutant BmSPI38 proteins against subtilisin (**A**), proteinase K (**C**), elastase (**E**), trypsin (**G**), and chymotrypsin (**I**), respectively. The inhibitory activities of the mutant BmSPI38 proteins against subtilisin (**B**), proteinase K (**D**), elastase (**F**), trypsin (**H**), and chymotrypsin (**J**) at the indicated molar ratio of inhibitor and protease. Error bars represent the standard error of the mean (*n* = 3). Different letters “a–c” indicate a significant difference between groups (*p* < 0.05), and one identical letter indicates no significant difference between groups (*p* < 0.05).

To further compare the intrinsic inhibitory activity of P1 mutants of BmSPI38, different molar concentrations of protease inhibitors were incubated with subtilisin, proteinase K, and elastase, respectively, and the residual enzyme activities were measured. With the increase in BmSPI38, BmSPI38(G54R), and BmSPI38(G54K) concentrations, the residual enzyme activities of subtilisin, proteinase K, and elastase gradually decreased, indicating that all of them could strongly inhibit the activities of the above three proteases (Figure 6A,C,E). Overall, the replacement of Gly at the P1 site with Arg or Lys resulted in a significantly reduced inhibition of BmSPI38 against subtilisin (Figure 6A,B), proteinase K (Figure 6C,D), and elastase (Figure 6E,F). Among them, the inhibitory capacity of BmSPI38(G54R) on subtilisin, proteinase K, and elastase was significantly lower than that of BmSPI38(G54K). At the same time, we also compared the ability of BmSPI38 P1 mutants to inhibit trypsin and chymotrypsin. As the molar ratio of inhibitor to protease increased, BmSPI38 did not show any inhibition towards trypsin and chymotrypsin (Figure 6G,I). BmSPI38(G54R) and BmSPI38(G54K) obtained strong trypsin inhibitory activity (Figure 6G) and weak chymotrypsin inhibitory activity (Figure 6I). The inhibitory capacity of BmSPI38(G54K) towards trypsin was significantly stronger than that of BmSPI38(G54R), while its inhibitory capacity towards chymotrypsin was significantly weaker than that of BmSPI38(G54R) (Figure 6G–J). The above results suggested that replacing the P1 residue with a strong basic amino acid could not only reduce the intrinsic activity of BmSPI39, but also alter its inhibitory specificity.

### 2.7. Comparison of the Inhibitory Ability of Mutant BmSPI39 Proteins to Different Serine Proteases

**Figure 7:**
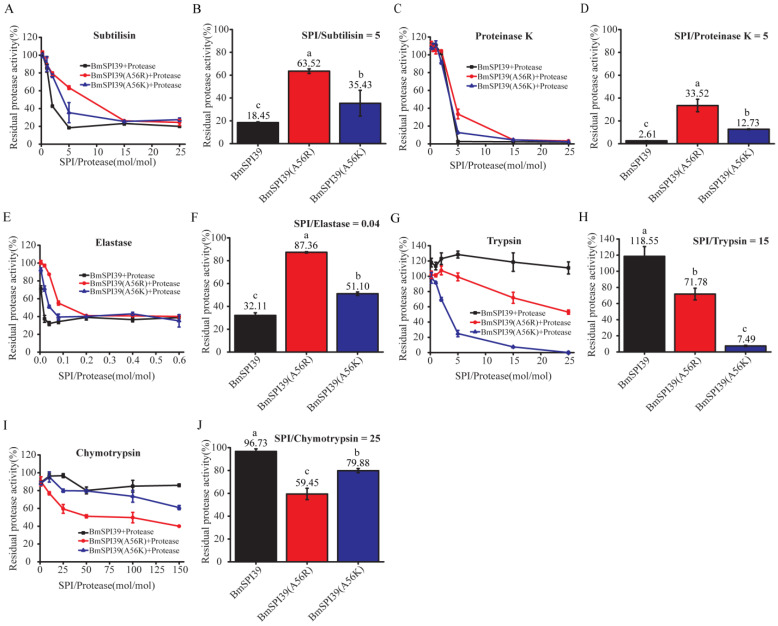
Comparison of the inhibitory ability of mutant BmSPI39 proteins to different serine proteases. Inhibitory effects of increasing concentrations of mutant BmSPI39 proteins against subtilisin (**A**), proteinase K (**C**), elastase (**E**), trypsin (**G**), and chymotrypsin (**I**), respectively. The inhibitory activities of the mutant BmSPI39 proteins against subtilisin (**B**), proteinase K (**D**), elastase (**F**), trypsin (**H**), and chymotrypsin (**J**) at the indicated molar ratio of inhibitor and protease. Error bars represent the standard error of the mean (*n* = 3). Different letters “a–c” indicate a significant difference between groups (*p* < 0.05), and one identical letter indicates no significant difference between groups (*p* < 0.05).

To further investigate the effect of replacing Ala at the P1 position with a strong basic amino acid on the intrinsic activity of BmSPI39, the protease inhibitors with different molar concentrations were incubated with subtilisin, proteinase K, or elastase, respectively, and the residual enzyme activities were determined. With the increase in the molar ratio of inhibitor to protease, the residual enzyme activities of subtilisin (Figure 7A), proteinase K (Figure 7C), and elastase (Figure 7E) gradually decreased, indicating that BmSPI39, BmSPI39(A56R), and BmSPI39(A56K) could strongly inhibit the activities of the above three proteases. The inhibitory activity of BmSPI39(A56R) towards the above three proteases was significantly lower than that of BmSPI39(A56K), while the activity of BmSPI39(A56K) was significantly lower than that of wild-type BmSPI39 (Figure 7B,D,F). Meanwhile, we also compared the ability of BmSPI39, BmSPI39(A56R), and BmSPI39(A56K) to inhibit trypsin and chymotrypsin. Wild-type BmSPI39 had no trypsin inhibitory activity, but showed very weak chymotrypsin inhibitory activity. After replacement of Ala 56 with Arg or Lys, the mutant protein of BmSPI39 acquired strong trypsin inhibitory activity and weak chymotrypsin inhibitory activity (Figure 7G–J). BmSPI39(A56K) showed significantly stronger inhibition against trypsin than BmSPI39(A56R), but significantly weaker inhibition against chymotrypsin than BmSPI39(A56R) (Figure 7G–J). The results presented above indicated that substitution of P1 residues with strong basic amino acids not only reduced the intrinsic activity of BmSPI39, but also enabled the mutant protein to acquire the inhibitory activity against trypsin and chymotrypsin.

### 2.8. P1 Mutants of BmSPI38 and BmSPI39 Have Extremely High Acid–Base and Thermal Stability

**Figure 8:**
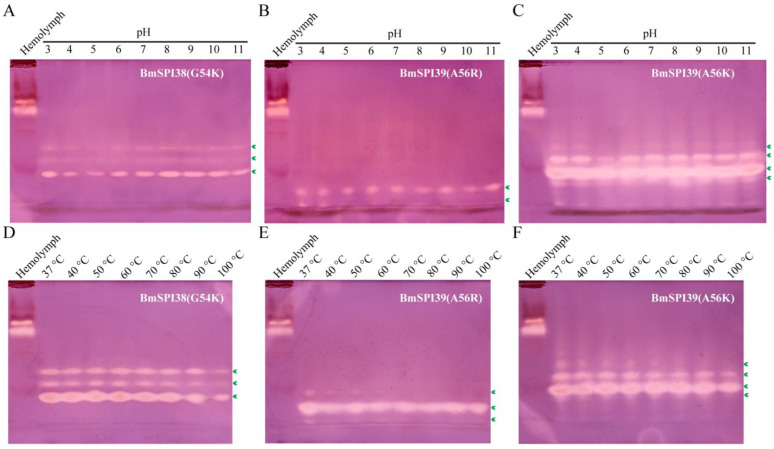
P1 mutants of BmSPI38 and BmSPI39 exhibited strong acid–base and thermal stability. Effect of pH on the activities of BmSPI38(G54K) (**A**), BmSPI39(A56R) (**B**), and BmSPI39(A56K) (**C**). Effect of temperature on the activities of BmSPI38(G54K) (**D**), BmSPI39(A56R) (**E**), and BmSPI39(A56K) (**F**). The inhibitory activity of purified mutant proteins on trypsin after treatment with different pH or temperatures can be assessed by in-gel activity staining. *B. mori* hemolymph from day 5 fifth-instar larvae was used as positive control. Green arrows indicate the inhibitory-active bands of the P1 mutants against trypsin.

Previous studies have confirmed that wild-type BmSPI38 and wild-type BmSPI39 have extremely high acid–base stability and thermal stability [11,12]. To explore the effect of P1 residue substitution on the physicochemical properties of BmSPI38 and BmSPI39, BmSPI38(G54K), BmSPI39(A56R), and BmSPI39(A56K) treated with different pH or temperature were separated by alkaline Native PAGE and stained for activity in the gel. The results showed that BmSPI38(G54K), BmSPI39(A56R), and BmSPI39(A56K) had no significant changes in the strength of the active bands against trypsin in the range of pH 3 to 11, indicating that they all had extremely high acid–base stability (Figure 8A–C). The inhibitory activity of BmSPI38(G54K) did not change significantly after treatment at 37–90 °C, but decreased slightly after treatment at 100 °C for 10 min (Figure 8D). BmSPI39(A56R) and BmSPI39(A56K) still maintained strong inhibitory activities against trypsin after treatment at 37–100 °C (Figure 8E,F). The above results showed that BmSPI38(G54K), BmSPI39(A56R), and BmSPI39(A56K) have extremely high thermal stability.

## 3. Discussion

Elastase is a serine protease with broad specificity that hydrolyzes connective tissue components such as elastin, proteoglycan, fibronectin, and collagen I, II, III, and IV, etc. Elastin is the major component of elastic fibers. Elastic fibers mainly exist in ligaments and vascular walls, endowing tissues with elasticity and tensile capacity, and are important components of tissues and organs such as skin, lungs, large arteries, and ligaments. The excessive production of elastase can lead to different degrees of tissue damage, and can cause or aggravate emphysema [31,32], acute respiratory distress syndrome [33], pancreatitis [34], rheumatoid arthritis [35,36], chronic bronchitis [37], nephritis [38], atherosclerosis [39,40], fatty liver [41], autoimmune diabetes [42], and other diseases, which seriously endanger human health. Elastase can also destroy collagen fibers and the basement membrane layer of tissues, thus causing cancer [43,44]. In addition, elastase can hydrolyze the elastin fibers of the skin, leading to skin wrinkling or sagging, and inhibiting the activity of elastase can prevent skin aging [45,46]. Elastase inhibitors can effectively inhibit the activity of elastase, and have important application value in the research and development of anti-inflammation, anti-tumor, and anti-skin aging drugs [47,48,49,50].

Silk protein has broad application prospects in many fields due to its unique characteristics of biocompatibility, biodegradability, self-assembly, mechanical stability, controllable structure, and morphology [51,52]. The silk cocoon extracts of *Antheraea assamensis*, *B. mori*, and *Philosamia ricini* have a certain inhibitory effect on porcine pancreatic elastase [50]. The treatment of ultraviolet-irradiated human skin fibroblasts with cocoon silk extracts can not only down-regulate the gene expressions of proinflammatory cytokines and matrix metalloproteinase-1, but also enhance the production of collagen, thus preventing skin photoaging caused by ultraviolet radiation [50]. Studies have shown that there are abundant protease inhibitors in *B. mori* silk, and TIL protease inhibitors represented by BmSPI39 and BmSPI38 enter the cocoon layer during the process of silk secretion, which endows the cocoon with strong anti-microbial protease hydrolysis properties [13,14,53,54]. The present study confirmed that BmSPI38 and BmSPI39 can strongly inhibit the activity of porcine pancreatic elastase, which was the first time the inhibitory activity of silkworm protease inhibitors against elastase was clarified (Figure 1). We speculated that the anti-elastase activity of cocoon silk extract might be closely related to the abundance of BmSPI38 and BmSPI39 in cocoon silk. As potent inhibitors of elastase, BmSPI38 and BmSPI39 not only provide a powerful tool for studying the relationship between the structure and function of elastase, but also have important research value in the fields of biomedicine and skin care. However, as a potential template for anti-inflammatory and anti-aging leading drugs, in vivo evaluation is essential.

It is found that BmSPI38 and BmSPI39 are very different from typical TIL protease inhibitors in structure and activity [11,12]. The second and sixth cysteines are absent in the TIL domain of BmSPI38 and BmSPI39. They cannot inhibit the activities of mammalian proteases such as trypsin and chymotrypsin but have strong microbial protease inhibitory activities. BmSPI38 and BmSPI39 with unique structure and activity may be good models for studying the relationship between the structure and function of small-molecule TIL-type protease inhibitors, which can provide a basis or reference for the activity and specificity modification of such inhibitors, and promote the production and application of TIL-type inhibitors. The simultaneous introduction of conserved Cys^2nd^ and Cys^6th^ resulted in a dramatic decrease in the inhibitory activities of BmSPI38 and BmSPI39 against subtilisin and proteinase K, but failed to alter their inhibitory specificity [21]. Obviously, there should be other amino acid sites involved in determining the inhibitory specificity of BmSPI38 and BmSPI39. Studies have shown that the P1 residue in the reactive center is usually one of the key sites that determines the inhibitory specificity of serine protease inhibitors [22,23,24,25,26]. In this study, the P1 residues of BmSPI38 and BmSPI39 were replaced by amino acids with different properties by site-directed mutagenesis, in order to explore the effect of P1 sites on the inhibitory activity and specificity of BmSPI38 and BmSPI39.

Studies have shown that different P1 residues may affect the conformation of protease inhibitors [55,56,57]. However, the P1 mutants of BmSPI38 and BmSPI39 still had inhibitory activity. This study found that all mutants of BmSPI38 and BmSPI39 could inhibit the activity of elastase. Except for BmSPI38(G54C), other mutants of BmSPI38 and BmSPI39 retained inhibitory activity against subtilisin (Figure 3 and Figure 4). This may reflect the broad specificity of elastase and subtilisin because the pockets of their active sites are large enough. Studies have shown that *B. mori* fungal protease inhibitor-F (BmFPI-F) has similar sequence characteristics and inhibitory activities with BmSPI38 and BmSPI39, and only has one TIL domain containing eight cysteine residues, which can inhibit the activities of microbial proteases such as subtilisin, proteinase K, and *A. melleus* protease [58]. When the P1 residues (Thr29) of BmFPI-F were replaced by six different amino acids (Glu, Arg, Gly, Leu, Met, and Phe), all the mutant proteins still retained the ability to inhibit subtilisin [59]. The saturation mutation of Met at the P1 position of *Streptomyces* subtilisin inhibitor (SSI) was carried out, and all mutants also showed different degrees of subtilisin inhibitory activity [60]. These findings are generally consistent with our results. Although almost all mutant proteins retained the inhibitory activities against subtilisin and elastase, the P1 residue substitution could greatly affect the strength of the intrinsic activity of BmSPI38 and BmSPI39. In general, when Gly54 in BmSPI38 and Ala56 in BmSPI39 were replaced with Gln, Ser, or Thr, their inhibitory activities against subtilisin and elastase were significantly enhanced. However, replacing P1 residues in BmSPI38 and BmSPI39 with Ile, Trp, Pro, or Val severely weakened their inhibitory activities against subtilisin and elastase. It has been reported that SSI mutants with P1 residues of polar amino acids Gln, Ser, or Thr have extremely strong subtilisin inhibitory activity, while mutants with P1 residues of Pro, Ile, Gly, or Val have weak inhibitory activity against subtilisin [60]. The replacement of Thr at the P1 position of BmFPI-F with Gly resulted in a greatly reduced [56] potency against subtilisin [59], which was exactly verified by the fact that replacing Gly at P1 with Thr could enhance the inhibitory activity of BmSPI38 against subtilisin.

Trypsin, chymotrypsin, and elastase are serine proteases with high sequence and structural similarity, but with different substrate specificity [61]. Trypsin is one of the endopeptidases that selectively hydrolyzes the peptide bond formed by the carboxyl group of the basic amino acid Arg or Lys in proteins. This is because trypsin has an open acidic S1 pocket, and the conserved Asp189 at the bottom of the S1 pocket can form a salt-bridge interaction with arginine or lysine at the P1 position of the substrate [62]. It has been shown that serine protease inhibitors whose P1 residues in the reactive center are a basic amino acid tend to inhibit trypsin. CmPI II is a Kazal protease inhibitor isolated from Caribbean snail *Cenchritis muricatus*, which can inhibit not only trypsin activity, but also subtilisin and elastase activity [24]. The substitution of Arg at the P1 site of CmPI II with Ala not only abolished its trypsin inhibitory activity, but also increased its inhibitory activity against subtilisin and elastase [24]. SSI mutants with P1 residues replaced by Arg and Lys obtained inhibitory activity against trypsin, while mutants with Tyr and Trp gained inhibitory activity against chymotrypsin [63]. The winged bean chymotrypsin inhibitors (WCI) belong to the Kunitz family, and replacing Leu at P1 with Arg can transform WCI(L56R) into a potent trypsin inhibitor [22]. This rule also applies to TIL-type protease inhibitors. In this study, it was found that replacing the P1 residues of BmSPI38 and BmSPI39 with Arg or Lys could not only reduce their inhibitory activities against subtilisin and elastase, but also enable the mutant proteins to obtain strong trypsin inhibitory activity and weak chymotrypsin inhibitory activity (Figure 5, Figure 6 and Figure 7). Egf1.0, a TIL-type protease inhibitor in *Microplitis demolitor* bracovirus, has only eight conserved cysteines in the TIL domain. The mutation of the P1 residue of Egf1.0 from Arg to Ala resulted in the loss of its inhibitory activity against prophenoloxidase activating proteinase-3 (PAP3, belonging to trypsin) [64]. *Cotesia vestalis* teratocytes can secrete a TIL-type protease inhibitor CvT-TIL, which can strongly inhibit the activation of prophenoloxidase in the hemolymph of the host insect by interacting with PAP3 of the phenoloxidase (PO) cascade pathway [65]. CvT-TIL also has a TIL domain containing eight conserved cysteine residues, and its P1 residue is Arg, which can not only inhibit the activity of subtilisin, but also inhibit elastase and trypsin to some extent [65]. It should be pointed out that BmSPI38(G54R), BmSPI38(G54K), BmSPI39(A56R), BmSPI39(A56K), and CvT-TIL have similar sequence characteristics. They all have a TIL domain containing eight cysteines, and P1 residues are strongly basic amino acids. BmSPI38(G54R), BmSPI38(G54K), BmSPI39(A56R), and BmSPI39(A56K) all showed much better inhibition of trypsin, subtilisin, and elastase than CvT-TIL. Can BmSPI38(G54R), BmSPI38(G54K), BmSPI39(A56R), and BmSPI39(A56K) inhibit the PO cascade in insects? What is the specific mechanism by which they inhibit the PO cascade? Can P1 mutants of BmSPl38 and BmSPl39 be used as target molecules to improve the virulence of biopesticides? All of these scientific questions need to be answered urgently.

It should be noted that the mutant proteins of BmSPI38 and BmSPI39 cannot be excluded from acquiring other new activities and functions due to the sensitivity of in-gel activity staining of protease inhibitor, and the limited types of proteases tested. For example, no inhibitory bands of BmSPI38(G54R), BmSPI38(G54K), BmSPI39(A56R), and BmSPI39(A56K) against chymotrypsin were detected by in-gel activity staining, while the protease inhibition assays confirmed that the above four mutants had weak inhibitory activities of chymotrypsin. Because BmSPI38(G54R) has relatively weak inhibitory activity against trypsin, only the physicochemical properties of BmSPI38(G54K), BmSPI39(A56R), and BmSPI39(A56K) with strong activity were investigated in this study. Similar to the wild-type BmSPI38 and BmSPI39 [11,12], the mutants with Arg or Lys at the P1 position have extremely high acid–base stability and thermal stability, which may be related to the abundant disulfide bonds in BmSPI38 and BmSPI39.

The results presented above indicate that the amino acid properties of P1 residues play an important role in the inhibitory activity and specificity of BmSPl38 and BmSPl39, which may be related to the conformational changes around the reactive center. Future studies should be carried out to obtain more mutants, reveal the structure–function relationship between BmSPl38, BmSPl39, and their target proteases, and obtain more potent and specific inhibitors using BmSPl38 and BmSPl39 as scaffolds for biomedicine and agroforestry pest control.

## 4. Materials and Methods

### 4.1. Escherichia coli Strains, Plasmids, and Reagents

*E. coli* Trans-T1, BL21(DE3), and Origami 2(DE3) were purchased from TransGen Biotech (Beijing, China), Sangon Biotech (Shanghai, China) and Invitrogen (Carlsbad, CA, USA), respectively. *TransStart^®^ FastPfu* DNA polymerase (*FastPfu*), *TransStart^®^ FastPfu* Fly DNA polymerase (*FastPfu* Fly) and *EasyPfu* DNA Polymerase (*EasyPfu*) were purchased from TransGen Biotech. Proteinase K from *Tritirachium album* limber and elastase from the porcine pancreas were purchased from Roche (Mannheim, Germany) and Sangon Biotech, respectively. Subtilisin A from *Bacillus licheniformis*, trypsin and α-chymotrypsin from the bovine pancreas, N-acetyl-D,L-phenylalanine-β-naphthylester (APNE) and Fast Blue B Salt were purchased from Sigma-Aldrich (St. Louis, MO, USA). Fluorescein isothiocyanate (FITC)-labeled casein was purchased from Thermo Fisher Scientific (Waltham, MA, USA). Recombinant expression plasmids *BmSPI38*-p28 and *BmSPI39*-p28 were preserved by the College of Biological Science and Engineering, Shaanxi University of Technology.

### 4.2. Expression Vector Construct of the P1 Mutants

According to the manual of the QuikChange II XL Site-Directed Mutagenesis Kit (Agilent, Santa Clara, CA, USA), 19 pairs of mutagenic primers were designed for site-directed saturation mutagenesis at the P1 position of BmSPI38 and BmSPI39, respectively. The templates, desired mutations, DNA polymerases, and primer sequences involved in P1-site mutations of BmSPI38 and BmSPI39 are shown in Table 1 and Table 2, respectively. *BmSPI38*-p28 and *BmSPI39-p28* recombinant expression plasmids were transferred into dam^+^ *E. coli* strain Trans1-T1. After overnight culture at 37 °C 220 r/min, methylated *BmSPI38*-p28 and *BmSPI39*-p28 plasmids were extracted again. Then, the *BmSPI38*-p28 or *BmSPI39*-p28 plasmid was used as a template for PCR amplification with *FastPfu* DNA polymerase to introduce target mutations. The PCR products were purified using *EasyPure^®^* PCR Purification Kit (TransGen Biotech) or *EasyPure^®^* Quick Gel Extraction Kit (TransGen Biotech), and then digested with the *Dpn* I restriction endonuclease (Thermo Fisher Scientific) at 37 °C for 30 min to completely remove the methylated template DNA. The *Dpn* I digested products were transformed into Trans1-T1 competent cells, and the monoclones were selected for sequencing verification. As shown in Table 1 and Table 2, the mutants that failed in the first round of PCR amplification can be obtained by changing the DNA polymerases (*FastPfu* Fly or *EasyPfu*) or templates (the mutant expression plasmid that has been successfully constructed) in the PCR. Finally, all the mutant expression vectors were constructed.

### 4.3. Expression and Purification of the Mutants

The expression plasmids of the P1 mutants of BmSPI38 and the expression plasmids of the P1 mutants of BmSPI39 were transferred into *E. coli* Origami 2(DE 3) and BL21(DE3) competent cells for induced expression, respectively. When the OD_600_ of the culture reached 0.6–1.0, protein expression was induced using IPTG at a final concentration of 0.2 mmol/L at 37 °C for 5 h or at 16 °C for 20 h. The *E. coli* cells were harvested by centrifugation at 6000× *g* for 30 min, washed, and suspended with binding buffer (20 mmol/L Tris-HCl, 500 mmol/L NaCl, pH 7.9). The *E. coli* cells were lysed by sonication, and bacterial precipitates and supernatants were collected by centrifugation at 16,000× *g* for 30 min. The expressions of mutant proteins were analyzed by 16.5% SDS-PAGE. The mutated proteins were purified with Ni^2+^-NTA (nitrilotriacetic acid) affinity chromatography (Sangon Biotech, Shanghai, China) as described for the wild-type proteins [11,12]. The lysate supernatant was loaded onto a 1 mL Ni^2+^-NTA affinity chromatography column. It was then washed and eluted sequentially by binding buffer supplemented with 20, 50, 100, and 400 mmol/L imidazole. The eluted fraction enriched with the target protein was pooled, and the imidazole was removed by over-night dialysis at 4 °C in binding buffer. The second round of immobilized-nickel affinity chromatography was performed. The purified mutant proteins were finally collected and dialyzed to 10 mmol/L PBS buffer (pH 7.4) for activity detection.

### 4.4. In-Gel Activity Staining of Protease Inhibitor

In-gel activity staining of protease inhibitor was performed as previously described, with a slight modification [11,66]. After separating using a 10% alkaline Native PAGE, the gels were placed in 5 mg/mL elastase, 0.07 mg/mL subtilisin A, 0.07 mg/mL proteinase K, 0.07 mg/mL trypsin, or 0.07 mg/mL chymotrypsin solution, and incubated in the dark for 30 min at 37 °C, with shaking at 45 rpm. After recovering the protease solution, the gels were washed with ddH_2_O, and then were allowed to stand in the dark at 37 °C for 30 min. The gels were stained for 15 min at 37 °C in the dark with a mixture of substrate (20 mg APNE dissolved in 10 mL of N,N′-dimethylformamide) and a staining solution (100 mg Fast Blue B Salt dissolved in 100 mL of 0.1 mol/L pH 8.0 Tris-HCl buffer containing 20 mmol/L CaCl_2_) at a ratio of 1:10. The gels were stained fuchsia due to the diazotization-coupling reaction of β-naphthol that was produced by protease hydrolyzed substrates (APNE) on the gel. Due to protease inhibition, the positions of protease inhibitors will not be stained and will be shown as white bands.

### 4.5. Protease Inhibition Assays

Depending on the hydrolytic capacity of different serine proteases to FITC-casein substrate, 3 pmol of subtilisin, 3 pmol of proteinase K, 750 pmol of elastase, 15 pmol of trypsin, or 15 pmol of chymotrypsin were used per reaction (per well) in the protease inhibition assay. To determine whether P1 residue replacement could alter the inhibitory specificity of BmSPI38 and BmSPI39, a relatively high dose of the protease inhibitors was incubated with the specified proteases. The molar ratios of protease inhibitor to subtilisin, proteinase K, elastase, trypsin, and chymotrypsin were set as follows: 15:1, 15:1, 0.2:1, 150:1, 150:1. In order to further compare the inhibitory capacity of the mutant proteins on different proteases, different molar concentrations of protease inhibitors were incubated with the specified proteases. The molar ratios of protease inhibitors to subtilisin or proteinase K were set as 0.2, 0.5, 1, 2, 5, 15, and 25. The molar ratios with elastase were set as 0.004, 0.02, 0.04, 0.08, 0.2, 0.4, and 0.6. The molar ratios with chymotrypsin were set as 1, 10, 25, 50, 100, and 150. The molar ratio of BmSPI38 mutants to trypsin were set as 1, 5, 25, 55, 100, and 150; the molar ratio of BmSPI39 mutants to trypsin were set as 0.2, 0.5, 1, 2, 5, 15, and 25. The protease inhibitor and protease were mixed and supplemented with 0.1 mol/L Tris-HCl (pH 7.5) to 100 μL, and then incubated at 37 °C for 30 min. Then, 100 μL FITC-casein was added and incubated at 37 °C for 60 min in darkness. The fluorescence intensity was measured at the excitation and emission wavelengths of 485/535 nm, and the residual enzyme activity was calculated. The inhibitory activity of the protease inhibitor against protease was assessed by the following formula: residual enzyme activity% = enzyme activity of experimental group/enzyme activity of control group × 100%. In the control group, the protease inhibitor was replaced by an equal volume of 10 mmol/L PBS buffer (pH 7.4).

### 4.6. Acid–Base and Thermal Stability Analysis

Equal masses of purified mutant proteins were mixed with Britton–Robinson buffer of different pH values (pH 3–11) and incubated at room temperature for 24 h. The purified mutant proteins were treated at different temperatures (37 °C, 40 °C, 50 °C, 60 °C, 70 °C, 80 °C, 90 °C, and 100 °C) for 10 min. Then, the treated protein samples were separated using 10% alkaline Native PAGE. Finally, the activities of mutant proteins were analyzed by in-gel activity staining.

### 4.7. Statistical Analysis

All statistical analyses of the data were performed using the Data Processing System (DPS) software version 9.01. Statistically significant differences were assessed by one-way analysis of variance (ANOVA). The error bar represents the standard error of the mean (*n* = 3). Marks with different lowercase letters “a to d” indicate significant differences between treatment groups at *p* < 0.05, while marks with one identical letter indicate no significant differences between treatment groups at *p* < 0.05.

## 5. Conclusions

In conclusion, this study demonstrated, for the first time, that silkworm protease inhibitors BmSPI38 and BmSPI39 can strongly inhibit the activity of elastase, and confirmed that the replacements of P1 residues not only greatly affect the intrinsic activity of BmSPI38 and BmSPI39, but also alter their inhibitory specificity when P1 residues are replaced with strong basic amino acids. This not only provides a new perspective and idea for the exploitation and utilization of BmSPI38 and BmSPI39 in biomedicine and pest control, but also provides a basis or reference for the activity and specificity modification of TIL-type protease inhibitors.

## Figures and Tables

**Table 1:** Mutagenic primers at P1 position of BmSPI38.

Mutants	Templates	Desired Mutations	DNA Polymerases	Primer Sequences ^a^
*BmSPI38(G54E)*	*BmSPI38*	G54E	*FastPfu*	F: 5′-CGCTTGCGTC**GAA**GCCCTCATTCAAACC-3′ R: 5′-TGAATGAGGGC**TTC**GACGCAAGCGGTG-3′
*BmSPI38(G54D)*	*BmSPI38*	G54D	*FastPfu*	F: 5′-CCGCTTGCGTC**GAT**GCCCTCATTCAAACC-3′ R: 5′- GTTGAATGAGGGC**ATC**GACGCAAGCGGTG-3′
*BmSPI38(G54R)*	*BmSPI38(G54S)*	S54R	*FastPfu*	F: 5′-ACCGCTTGCGTC**CGC**GCCCTCATTC-3′ R: 5′-AATGAGGGC**GCG**GACGCAAGCGGTG-3′
*BmSPI38(G54K)*	*BmSPI38*	G54K	*FastPfu*	F: 5′-ACCGCTTGCGTC**AAA**GCCCTCATTCAAACC-3′ R: 5′-GTTTGAATGAGGGC**TTT**GACGCAAGCGGTG-3′
*BmSPI38(G54H)*	*BmSPI38(G54D)*	D54H	*FastPfu*	F: 5′-ACCGCTTGCGTC**CAT**GCCCTCATTCAAAC-3′ R: 5′-TGAATGAGGGC**ATG**GACGCAAGCGGTG-3′
*BmSPI38(G54M)*	*BmSPI38*	G54M	*FastPfu*	F: 5′-CCGCTTGCGTC**ATG**GCCCTCATTCAAACC-3′ R: 5′-GTTTGAATGAGGGC**CAT**GACGCAAGCGGTG-3′
*BmSPI38(G54L)*	*BmSPI38*	G54L	*FastPfu*	F: 5′-CACCGCTTGCGTC**CTG**GCCCTCATTC-3′ R: 5′-GAATGAGGGC**CAG**GACGCAAGCGGTG-3′
*BmSPI38(G54I)*	*BmSPI38*	G54I	*FastPfu*	F: 5′-CCGCTTGCGTC**ATT**GCCCTCATTCAAACC-3′ R: 5′-GTTTGAATGAGGGC**AAT**GACGCAAGCGGTG-3′
*BmSPI38(G54F)*	*BmSPI38(G54I)*	I54F	*FastPfu*	F: 5′-CACCGCTTGCGTC**TTT**GCCCTCATTCAAAC-3′ R: 5′-TTTGAATGAGGGC**AAA**GACGCAAGCGGTG-3′
*BmSPI38(G54W)*	*BmSPI38(G54C)*	C54W	*FastPfu* Fly	F: 5′-CCGCTTGCGTC**TGG**GCCCTCATTCAAACC-3′ R: 5′-TTTGAATGAGGGC**CCA**GACGCAAGCGGTG-3′
*BmSPI38(G54N)*	*BmSPI38(G54Y)*	Y54N	*FastPfu* Fly	F: 5′-CCGCTTGCGTC**AAC**GCCCTCATTCAAAC-3′ R: 5′-TGAATGAGGGC**GTT**GACGCAAGCGGTG-3′
*BmSPI38(G54Q)*	*BmSPI38(G54P)*	P54Q	*EasyPfu*	F: 5′-ACCGCTTGCGTC**CAG**GCCCTCATTCAAAC-3′ R: 5′-TGAATGAGGGC**CTG**GACGCAAGCGGTG-3′
*BmSPI38(G54Y)*	*BmSPI38*	G54Y	*FastPfu*	F: 5′-CACCGCTTGCGTC**TAC**GCCCTCATTCAAAC-3′ R: 5′-GTTTGAATGAGGGC**GTA**GACGCAAGCGGTG-3′
*BmSPI38(G54C)*	*BmSPI38*	G54C	*FastPfu*	F: 5′-CACCGCTTGCGTC**TGC**GCCCTCATTC-3′ R: 5′-GAATGAGGGC**GCA**GACGCAAGCGGTG-3′
*BmSPI38(G54S)*	*BmSPI38*	G54S	*FastPfu*	F: 5′-CCGCTTGCGTC**AGC**GCCCTCATTCAAAC-3′ R: 5′-GAATGAGGGC**GCT**GACGCAAGCGGTG-3′
*BmSPI38(G54T)*	*BmSPI38*	G54T	*FastPfu*	F: 5′-CCGCTTGCGTC**ACC**GCCCTCATTCAAAC-3′ R: 5′-TTGAATGAGGGC**GGT**GACGCAAGCGG-3′
*BmSPI38(G54A)*	*BmSPI38(G54P)*	P54A	*EasyPfu*	F: 5′-CCGCTTGCGTC**GCG**GCCCTCATTCAAAC-3′ R: 5′-TGAATGAGGGC**CGC**GACGCAAGCGGTG-3′
*BmSPI38(G54P)*	*BmSPI38*	G54P	*FastPfu*	F: 5′-CGCTTGCGTC**CCG**GCCCTCATTCAAAC-3′ R: 5′-AATGAGGGC**CGG**GACGCAAGCGGTG-3′
*BmSPI38(G54V)*	*BmSPI38(G54L)*	L54V	*FastPfu* Fly	F: 5′-ACCGCTTGCGTC**GTG**GCCCTCATTCAAAC-3′ R: 5′-TGAATGAGGGC**CAC**GACGCAAGCGGTG-3′

^a^ The mutated codons are shown in bold, and the mutated bases are underlined.

**Table 2:** Mutagenic primers at P1 position of BmSPI39.

Mutants	Templates	Desired Mutations	DNA Polymerases	Primer Sequences ^a^
*BmSPI39(A56E)*	*BmSPI39(A56D)*	D56E	*FastPfu*	F: 5′-CCAGCTGCGTA**GAA**GCATTGCTCCCAACATG-3′ R: 5′-TGGGAGCAATGC**TTC**TACGCAGCTGGTGTG-3′
*BmSPI39(A56D)*	*BmSPI39*	A56D	*FastPfu* Fly	F: 5′-CACCAGCTGCGTA**GAT**GCATTGCTCCCAAC-3′ R: 5′-GTTGGGAGCAATGC**ATC**TACGCAGCTGGTG-3′
*BmSPI39(A56R)*	*BmSPI39*	A56R	*FastPfu*	F: 5′-CAGCTGCGTA**CGC**GCATTGCTCCCAAC-3′ R: 5′-GGGAGCAATGC**GCG**TACGCAGCTGGTG-3′
*BmSPI39(A56K)*	*BmSPI39(A56L)*	L56K	*FastPfu* Fly	F: 5′-CACCAGCTGCGTA**AAG**GCATTGCTCGGAAC-3′ R: 5′-TTGGGAGCAATGC**CTT**TACGCAGCTGGTG-3′
*BmSPI39(A56H)*	*BmSPI39*	A56H	*FastPfu*	F:5′-CACCAGCTGCGTA**CAT**GCATTGCTCCCAAC-3′ R: 5′-GGGAGCAATGC**ATG**TACGCAGCTGGTGTG-3′
*BmSPI39(A56M)*	*BmSPI39(A56L)*	L56M	*FastPfu* Fly	F: 5′-CACCAGCTGCGTA**ATG**GCATTGCTCCCAAC-3′ R: 5′-TTGGGAGCAATGC**CAT**TACGCAGCTGGTG-3′
*BmSPI39(A56L)*	*BmSPI39*	A56L	*FastPfu*	F: 5′-CACCAGCTGCGTA**CTG**GCATTGCTCCCAAC-3 ′ R: 5′-GGGAGCAATGC**CAG**TACGCAGCTGGTGTG-3 ′
*BmSPI39(A56I)*	*BmSPI39*	A56I	*FastPfu*	F: 5′-CCAGCTGCGTA**ATT**GCATTGCTCCCAACATG-3′ R: 5′-TGGGAGCAATGC**AAT**TACGCAGCTGGTGTG-3′
*BmSPI39(A56F)*	*BmSPI39(A56I)*	I56F	*FastPfu*	F: 5′-CACCAGCTGCGTA**TTT**GCATTGCTCCCAAC-3′ R: 5′-TGGGAGCAATGC**AAA**TACGCAGCTGGTGTG-3′
*BmSPI39(A56W)*	*BmSPI39*	A56W	*FastPfu*	F: 5′-CACCAGCTGCGTA**TGG**GCATTGCTCCCAAC-3′ R: 5′-GGGAGCAATGC**CCA**TACGCAGCTGGTGTG-3′
*BmSPI39(A56N)*	*BmSPI39(A56Y)*	Y56N	*EasyPfu*	F: 5′-CACCAGCTGCGTA**AAC**GCATTGCTCCCAAC-3′ R: 5′-TTGGGAGCAATGC**GTT**TACGCAGCTGGTG-3′
*BmSPI39(A56Q)*	*BmSPI39(A56L)*	L56Q	*EasyPfu*	F:5′-CACCAGCTGCGTA**CAG**GCATTGCTCCCAAC-3′ R: 5′-TTGGGAGCAATGC**CTG**TACGCAGCTGGTG-3′
*BmSPI39(A56Y)*	*BmSPI39*	A56Y	*FastPfu*	F: 5′-CCAGCTGCGTA**TAC**GCATTGCTCCCAACATG-3′ R: 5′-TGGGAGCAATGC**GTA**TACGCAGCTGGTGTG-3′
*BmSPI39(A56C)*	*BmSPI39*	A56C	*FastPfu*	F: 5′-CCAGCTGCGTA**TGC**GCATTGCTCCCAAC-3′ R: 5′-GGGAGCAATGC**GCA**TACGCAGCTGGTG-3′
*BmSPI39(A56S)*	*BmSPI39*	A56S	*FastPfu*	F: 5′-CCAGCTGCGTA**AGC**GCATTGCTCCCAAC-3′ R: 5′-GGGAGCAATGC**GCT**TACGCAGCTGGTG-3′
*BmSPI39(A56T)*	*BmSPI39*	A56T	*FastPfu* Fly	F: 5′-CCAGCTGCGTA**ACC**GCATTGCTCCCAAC-3′ R:5′-GGGAGCAATGC**GGT**TACGCAGCTGGTG-3′
*BmSPI39(A56G)*	*BmSPI39*	A56G	*FastPfu*	F: 5′-CCAGCTGCGTA**GGC**GCATTGCTCCCAAC-3′ R: 5′-GGGAGCAAT**GCG**CCTACGCAGCTGGTG-3′
*BmSPI39(A56P)*	*BmSPI39*	A56P	*FastPfu*	F: 5′-CACCAGCTGCGTA**CCG**GCATTGCTCCC-3′ R: 5′-GGGAGCAATGC**CGG**TACGCAGCTGGTG-3′
*BmSPI39(A56V)*	*BmSPI39(A56L)*	L56V	*EasyPfu*	F: 5′-CACCAGCTGCGTA**GTG**GCATTGCTCCCAAC-3′ R: 5′-TTGGGAGCAATGC**CAC**TACGCAGCTGGTG-3′

^a^ The mutated codons are shown in bold, and the mutated bases are underlined.

## Data Availability

Not applicable.

## Sample Availability

The samples are available from the corresponding author upon reasonable request.
